# Psychometric properties of the Greek Version of the Traumatic Event Scale (TES) (Version A) among low-risk pregnant women

**DOI:** 10.1186/s40359-023-01152-z

**Published:** 2023-04-07

**Authors:** Pinelopi Varela, Aikaterini Lykeridou, Ioannis Zervas, Anna Deltsidou

**Affiliations:** 1 General Hospital of Athens “Alexandra”, Department of Midwifery, University of West Attica, Athens, Greece; 2 Department of Midwifery, University of West Attica, Athens, Greece; 3 Professor of Psychiatry and Psychosomatic Medicine, Head of the Women’s mental health and reproductive psychiatric clinic, National and Kapodistrian University of Athens Medical School, Eginition University Hospital, Athens, Greece

**Keywords:** Posttraumatic stress disorder, PTSD, Pre-traumatic stress, Traumatic event Scale, Psychometric properties, Validity, Reliability, Confirmatory factor analysis, Greece

## Abstract

**Background:**

The Traumatic Event Scale (TES) is one of the most often used instruments for the assessment of the Posttraumatic Stress Disorder (PTSD) symptomatology during pregnancy which is linked with adverse effects. The aim of the study was to assess the psychometric properties of the TES (version A) in a sample of Greek pregnant women.

**Methods:**

Two hundred one low risk pregnant women in their second or third trimester were invited to participate in the study. Participants completed a number of questionnaires including the Greek versions of TES-A, State-Trait Anxiety Inventory (STAI), Coping Orientations to Problems Experienced (Brief COPE), Perceived Stress Scale (PSS-10) and Edinburgh Postnatal Depression Scale (EPDS). Confirmatory factor analysis (CFA) was conducted in order to test how well the already TES-A five-factor model fits the data from Greece.

**Results:**

Participants’ average age was 34.2 years (SD = 4.3 years). Through CFA the already five-factor structure of the TES-A (Anticipation of trauma, Intrusion, Avoidance, Resignation, Hyperstimulation) was applied to our sample. All five factors were significantly and positively correlated with each other. All Cronbach’s alpha were over 0.7, indicating acceptable reliability of the factors. Relatively convergent validity, all factors of the Greek version of the TES-A were significantly associated with stress, anxiety, depression and coping strategies.

**Conclusion:**

The Greek version of TES-A is detected to be a valid and reliable instrument of prenatal Posttraumatic Stress Disorder (PTSD) symptomatology among low-risk Greek pregnant women.

## Background

Childbirth is a common part of life for most women, yet each birth experience is unique. During childbirth, women may perceive dangers to their lives or major injury, or there may be a real injury to the woman or her child. Childbirth is increasingly recognized as a potentially traumatic experience that can lead to Posttraumatic Stress Disorder (PTSD) [1].

According to Diagnostic and Statistical Manual (DSM-V), PTSD is characterized by the development of symptoms of intrusions, avoidance, negative alteration in cognition and mood, and alterations in arousal and reactivity. All eight DSM-V diagnostic criteria are required to be met in order to be diagnosed PTSD after any event. First of all, it must be determined that the event met the criteria for being traumatic (Criterion A). The symptoms of intrusive or disturbing memories must then be present (Criterion B), avoidance of memories or external reminders of events for an extended period of time (Criterion C), unfavorable alterations in cognition or mood that start or intensify soon after the event (Criterion D), marked changes in arousal or emotional reactivity after the event (Criterion E) that have persisted for over a month (Criterion F) and they had to have hampered the woman’s ability to perform in key areas of her daily life (Criterion G), and finally, not be attributable to substance use or medication (Criterion H). Individuals must have been subjected to real or threatened death, significant injury, or sexual violence in one of the following ways or more: direct, witnessed, indirect, repeated or extreme indirect exposure [2].

Childbirth is distinct from other traumatic experiences in that it is expected from the onset of the pregnancy. Despite the fact that such an event is anticipated, some parts of labor remain unknown, forcing some women to consider childbirth to be a danger. Surveys such as that conducted by Söderquist, Wijma, and Wijma showed that 2.3% of participants met full criteria for PTSD based on anticipated trauma of childbirth and 5.8% met criteria B, C and D, suggesting that pre-traumatic stress related to the threatening forthcoming delivery might exist [3]. According to existing research, following traumatic birth experiences, requests for an elective cesarean section during a later pregnancy were considered to be a kind of avoidance [4, 5]. Previous studies also have reported trauma reactions among women who had prolonged labors with severe pain or insufficient support [6]. Ayers and Pickering have suggested the importance of focusing on the cause of PTSD symptoms during pregnancy, as their research results demonstrated that among the 2.8% of women who met the criteria for postpartum PTSD at 6 weeks postpartum, had significant symptoms during pregnancy [7]. As it seems, a body of research has established the nature of childbirth-related PTSD and furthermore has pointed out the time of onset and its duration. So, PTSD can occur before or during pregnancy, or it can been developed during the perinatal period [8] and according to reports it lasts from conception to one year after childbirth [9].

Research activity has also attempted to establish the prevalence of prenatal PTSD. Its prevalence has been estimated from 0 to 35% [10, 11]. While according to a recent systematic review and meta-analysis the prevalence of PTSD in pregnancy was 3.3% in community samples and 18.95% in high-risk samples [12]. Differences in prevalence can be caused by a variety of factors, including sampling, measurement, and cultural context. These differences in prevalence are common in the epidemiology of mental health disorders [13].

Several studies have already documented the detrimental effects of prenatal PTSD on obstetric outcomes. Research findings have shown that women with antenatal PTSD are at increased risk of preterm birth [14, 15]. Furthermore, PTSD it is possible to have a detrimental influence on the couple’s relationship as well as the parent-child bond [16] and there are indications that it may also affect infant emotion regulation and development [17].

The relevance of identifying and screening for PTSD during the perinatal period has already been highlighted by previous authors of published research [12, 18]. Among the diagnostic measures used to identify PTSD are interviews and diagnostic self-report questionnaires [12], such as the Traumatic Event Scale (TES) [19]. Studies on prenatal PTSD symptoms frequently employ the TES [3, 20, 21].

TES version A was explicitly created to evaluate traumatic stress symptoms, related to impending labor. It includes all of the PTSD symptom criteria, was created in accordance with the DSM-IV criteria for PTSD and its psychometric properties showing a valid and reliable tool. The authors of the original version of the scale did not mention the performance of confirmatory or explanatory factor analysis [19]. However, a later study from France examined the psychometric properties of TES-A, and the factor analysis performed yielded five factors. In addition, the values of Cronbach’s alpha for the five factors ranged from 0,61 to 0,81, while the values of the Spearman-Brown ranged from 0,80 to 0,90 [22].

Although there is research on psychological symptoms during pregnancy in Greece [23–25] which is not just restricted to low-risk pregnancies [26], it is primarily concerned with evaluating and measuring depressive and anxiety symptoms. These symptoms have also been significantly associated with postpartum depression [24]. Additionally, research has demonstrated that Greek pregnant women who were at risk of depressive symptomatology had a lower quality of life than pregnant women who were not at risk. This is in addition to the finding that prenatal depressive symptoms were positively connected with an unpleasant occurrence during pregnancy [25]. According to the literature, an established risk factor for symptoms of depression and anxiety is exposure to traumatic events [27, 28]. Interestingly, it has also been found that among pregnant women, the trauma occurrence score was correlated with symptoms of PTSD, generalized anxiety, and depression [29]. Studies have demonstrated that in response to trauma exposure, the HPA axis altered activity may be a factor in the development of mood and anxiety symptoms [30, 31]. For pregnant women, various relationships between trauma exposure, HPA axis dysregulation, and depressive and anxiety symptoms have already been observed [32–34].

Therefore, based on the aforementioned, it seems sensible to simultaneously assess prenatal PTSD and its relationship to prenatal depressive and anxiety symptoms. In this way, the psychological profile of pregnant women can be better understood by health care providers. This will lead to more concentrated clinical and scientific activity. In Greece, there seems to be a gap in the assessment and measurement of prenatal PTSD, despite the research sensitivity of Greek researchers around prenatal psychology. In this regard, the Greek validation of the TES (version A) not only appears reasonable and essential for both scientific and research objectives, but it also represents a significant first step in the assessment of prenatal PTSD in the Greek pregnant community. Therefore, the aim of the present study was to assess the psychometric characteristics of TES (version A) among Greek pregnant women.

## Methods

### Translation of the TES (version A)

Following getting permission from the scale’s developer, (Professor Klaas Wijma) [19], the process of translation was started. There were four steps in the procedure; forward translation, synthesis of the translations, back translation and Expert Committee and submission of documentation to the developer.

### Pilot test of the TES (version A)

The scale’s test-retest reliability came up when the instrument was administrated to the same sample group of 30 pregnant women at different times. The interval between the two administrations was 20 days. The test–retest reliability (intraclass correlation coefficients, ICC) for TES version A ranged from 0.76 to 0.96 and Cronbach’s a reliability coefficient of five dimensions of the scale ranged from 0.62 to 0.87. The full details of the translation procedure and the pilot study have been published [35]. The Greek version of the TES (GrTES) version A was created after the accomplishment of the pilot study.

### Participants

The participants of the present study were pregnant women during their second or third trimester of pregnancy. The following were the inclusion criteria: low-risk pregnant women aged over 18 years with an adequate understanding of the Greek language. If a pregnant woman had a high-risk pregnancy, a twin or multiple pregnancy, a severe chronic condition, a psychiatric illness, or was taking psychiatric medication, she was excluded from the study.

### Procedure

From July 2020 to July 2021, the study was carried out in a public maternity hospital in Athens. The principal researcher approached pregnant women during their routine antenatal examination and invited them to participate in the study. Of the 240 invited women, 201 accepted the invitation to participate and signed an informed consent form. A booklet of questionnaires was provided to the participants, and they were instructed to fill it out and submit it to their following follow-up appointment. The booklet contained six self-administered questionnaires, one of which included demographic details, questions about mental health, and obstetric history. The descriptions of the rest of the questionnaires are provided below.

### Measures

The Traumatic Event Scale version A (TES-A):

The TES-A is a self-report questionnaire and has been developed especially to measure traumatic stress symptoms related to the forthcoming delivery. It was developed according to the DSM-IV criteria for PTSD and comprises all symptom criteria for PTSD. Four statements that are modifiable depending on the particular trauma of interest make up criterion A. Each statement is followed by four alternate responses: “not at all”, “somehow”, “much”, and “very much”. Criteria B, C, and D are the 17 sentences that follow criterion A and include the DSM-IV PTSD symptoms (i.e. intrusive thoughts, avoidance and numbing, and arousal). Participants rate the frequency of the symptoms indicated in the statements by choosing from one of four options: “never/not at all”, “rarely”, “sometimes” or “often”. Criterion F is assessed by the degree of the “severity” on a scale from 0 to 10 (not at all to extremely influenced) for every statement, expressing the extent to which the statement’s content has an impact on the participant’s everyday life. Criterion E (i.e. the duration of symptoms) is assessed by means of a 13-point scale, ranging from “less than 4 weeks” to “more than 12 months”. The original version’s Cronbach’s alpha was 0.84 and split-half reliability was 0.90 [19]. Participants were asked to fill in the Greek version of the TES-A (GrTES-A).

Coping Orientations to Problems Experienced (Brief COPE): This scale assesses dispositional or situation-specific coping by evaluating a number of dissimilar coping strategies used by people in general or in a specific scenario. The Brief COPE is a 28-item assessment of a person’s coping mechanisms for stress and problems. The items assess fourteen coping strategies and respondents rate them using a four-point Likert scale ranging from “not at all” to “very much” [36]. The Greek version of the Brief COPE scale has sufficient psychometric characteristics with a Cronbach’s alpha ranging from 0.48 to 0.93 [37].

The State-Trait Anxiety Inventory (STAI): The STAI is composed of two subscales; the State subscale measures anxiety at the moment of assessment, which might change over time, and the Trait subscale evaluates anxiety level as a persistent personal feature, which is stable over time. Twenty items in total, each of which is scored on a Likert scale from 1 to 4, consist each subscale. The total score ranges from 20 to 80, for each subscale, and higher scores indicate higher levels of anxiety.

[38]. The scale has been translated and validated in the Greek population and Cronbach’s alpha was found to be 0.93 for the state anxiety subscale and 0.92 for the trait anxiety subscale [39].

The Perceived Stress Scale (PSS-10): The 10-item PSS scale requests from respondents to rate the frequency of their feelings and thoughts in connection to situations and circumstances that occurred in the previous month to measure how stressful experiences are regarded. Each item receives a score on a five-point Likert scale (0 = never to 4 = very often). After reversing positive item scores and adding up all scores, total scores are calculated, which range from 0 to 40. A higher score indicates greater stress [40]. The Greek version of the PSS-10 presented satisfactory psychometric properties and a Cronbach’s alpha of 0.82 [41].

Edinburgh Postpartum Depression Scale (EPDS): The scale is composed of ten statements that describe depressive symptoms. It contains four potential answers, and each one is rated according to how severe or pervasive the symptom is. The responses are given a score between 0 and 3, and their sum is then determined [42]. The EPDS scale has been translated and validated in the Greek population, with the internal consistency reliability of the scale being excellent (Cronbach’s alpha = 0.9) [43].

### Statistical analysis

Quantitative variables were expressed as mean values (Standard Deviation) and as median (interquantile range), while qualitative variables were expressed as absolute and relative frequencies. Confirmatory factor analysis (CFA), with the maximum likelihood estimation method, was conducted in order to test how well the French version of the TES-A five-factor model fits the data from Greece. We used the chi-square by degrees of freedom ratio (χ^2^/df), the CFI, the TLI, the RMSEA and the SRMR as goodness-of-fit indices [44], and these parameters were considered adequate when χ^2^/df ≤ 2.0, CFI ≥ 0.90,TLI ≥ 0.90 RMSEA ≤ 0.05 and SRMR < 0.08 [45–47]. Internal consistency reliability was determined by the calculation of Cronbach’s α coefficient. Scales with reliabilities equal to or greater than 0.70 were considered acceptable. Concurrent validity of TES was assessed via Pearson’s correlations coefficients with EPDS, PSS-10, Brief-cope and STAI scales. Convergent validity was tested through intercorrelations (Pearson’s r) among the five TES factors. All reported p values are two-tailed. Statistical significance was set at p < .05 and analyses were conducted using STATA statistical software (version 13.0).

## Results

### Sociodemographic characteristics

Two hundred-one pregnant women, with an average age of 34.2 years, comprised the sample (SD = 4.3 years). Their characteristics are presented in Table 1. The majority of the sample was Greek (96.0%) and married/living with their partner (96.5%). More than half of the sample was university alumni (60.7%) and 59.7% were working full-time. Also, 52.7% of the women had monthly family income 1,000–3,000 euro. Almost one out of two women had children, with the percentage being 48.8%. Moreover, 29.9% had visited a specialist for psychological problems in the past and 5.5% had taken treatment for such a problem. Psychotherapy had done 22.4% of the sample and 40.8% had lived a stressful event during last year. Furthermore, 24.4% had been abused during childhood, 23.9% during adulthood and 23.9% during a visit to a health professional.

### Confirmatory factor analysis

Via CFA we examined the fitting of the French version of the TES-A 5-factor structure. Several indices assessing the degree to which the model fit the data were computed. As suggested by Byrne [48], we computed several alternative indices of fit, including the χ^2^ value and *df*, root-mean-square error of approximation (RMSEA), comparative fit index (CFI), Tucker Lewis Index (TLI), and Standardized Root Mean Square Residual (SRMR) (Table 2). The indexes were in acceptable ranges, that the 5-factor structure was acceptable. The first factor is named “Anticipation of trauma”, the second factor is called “Intrusion”, the third factor is named “Avoidance”, the fourth factor is called “Resignation” and the fifth factor is named “Hyperstimulation”.

### Internal consistency

Descriptive of TES items, item-total correlations and Cronbach’s a for each factor are presented in Table 3. All Cronbach’s alpha coefficients were above 0.7, indicating acceptable reliability. Also, the removal of any of the items did not improve the alpha coefficient of the correspondent factor, thus no item needed to be removed.

### Correlation coefficients between TES-A factors

Descriptives of all five factors are presented in Table 4, along with their correlation coefficients between them. All factors were significantly and positively correlated with each other.

### Convergent validity

Greater values in EPDS, PSS-10 and STAI scales are positively and significantly correlated with all TES-A factors (Table 5). Moreover, greater scores in Avoidance and Express negative feelings were significantly associated with greater values in all factors except for factor Avoidance. More behavioural disengagement was significantly associated with greater values in all factors except for factor Anticipation of trauma.

## Discussion

The current study aimed to contribute to the validation of the Greek version of the TES-A on a group of pregnant Greek women due to the lack of studies on its psychometric properties. The five-factor structure of the TES-A that emerged from a previous French published study [22], applied to our sample. Moreover, all five factors were significantly and positively correlated with each other. The fact that there is an agreement between study results from factor analyses of two different countries, raises the possibility that the TES-A is not unidimensional. In addition, this finding highlight how, despite cultural differences, PTSD symptoms in pregnant women around different countries seem to share certain basic factors. Therefore, it is possible that the TES-A can be used to compare samples from various countries. The final Greek version of TES-A consists of all 21 items from original version [19] and has the same 5-factor structure as determined in French population [22] (Anticipation of trauma, Intrusion, Avoidance, Resignation, Hyperstimulation).

The Greek version of TES-A (GrTES-A) proved adequate internal consistency, suggesting a reliable and vigorous scale since Cronbach’s alpha for each of the five factors was over 0.7. The findings of the present study seem to be consistent with the previous French study which found that Cronbach’s alpha for each of the five factors ranged from 0.61–0.81 [22]. This also accords with the earlier observation from the original study which showed that Cronbach’s alpha of the scale was 0.84 [19].

Previous studies on pre-traumatic stress used measures of anxiety, depression, and coping strategies in order to investigate if these psychological parameters can be act as risk factors or predictors for pre-traumatic stress. Their findings suggested that anxiety symptoms, depressive symptoms, and coping strategies can be risk factors or predictors for pre-traumatic stress [49, 50]. According to the above previous research findings, the use of measures of stress, anxiety, depression, and coping strategies in order to perform convergent validity of GrTES-A was a reasonable action. Thus, the present study found that with regard to convergent validity, all factors of GrTES-A were significantly associated with PSS-10, STAI and EPDS. Also, the majority of the factors were significantly associated with strategies for coping. These findings demonstrate that the TES-A correlates with other measures of stress, anxiety, depression, and coping strategies. The present findings seem to be consistent with the previous French-published study which also found that the TES positively correlated with measures of worry and anxiety [22].

The results of the present study show that the Greek version of the TES-A is characterized by good and acceptable psychometric properties. The GrTES-A can be an effective assessment and screening tool for pre-traumatic stress in Greek pregnant women, and it is simple to use in clinical practice for healthcare providers in perinatal care. The tool’s value resides in its ability to identify pregnant women exhibiting PTSD symptoms who either have a suspicious psychological profile and history or do not, by administrating it. The detection of PTSD symptoms gives health professionals the opportunity to intervene in the pregnant’s best interest, thus reducing the risk of future adverse effects of PTSD.

A number of limitations need to be noted regarding the present study. It is unsafe to generalize the findings to the entire nation’s pregnant population, because the sample was drawn from a sizable Greek city. Since the women who participated in the study get regular prenatal care at the study hospital, the findings cannot be applied to women who do not get regular prenatal care. Moreover, we only included low risk pregnant women, thus the findings might not be relevant to high-risk pregnant women. Notwithstanding these limitations, this is the first research attempt to assess the reliability and validity of the Greek version of the TES-A among Greek low-risk pregnant women. This scale can be a powerful and effective instrument in the hands of healthcare experts to first identify pregnant women who are vulnerable and at risk and then take appropriate action. More research is needed to determine the multidimensionality of the TES-A. The performing of factor analysis from future studies will reveal if indeed the scale is not unidimensional. Additionally, by doing so, researchers in this scientific field will have a clearer understanding of the structure of the factors regarding PTSD symptomatology in pregnancy. Furthermore, country-to-country comparisons of factor analysis would be interesting. It is also recommended that future research be undertaken in samples of high-risk pregnancies. Researchers will then be able to contrast data from high-risk pregnancies with results from low-risk pregnancies in a more detailed manner.

## Conclusions

The present study presented a valid and reliable Greek version of TES-A with the inclusion of all items and resulting in five factors. For research and clinical applications in low-risk Greek pregnant women, the Greek version of the TES-A was shown to be appropriate. As a result, it is recommended to utilize the tool to assess PTSD symptoms in Greek pregnant women.

**Table 1:** Sample characteristics

	N (%)
Age, mean (SD)	34.2 (4.3)
Nationality	
Greek	193 (96.0)
Other	8 (4.0)
Married/ Living with partner	194 (96.5)
Educational level	
High school at most	57 (28.4)
University	122 (60.7)
Postgraduate degree	22 (10.9)
Occupation	
Full time employee	120 (59.7)
Part time employee	24 (11.9)
Free-lancer	10 (5.0)
Unemployed	29 (14.4)
Household	18 (9.0)
Monthly family income	
Up to 1.000 €	74 (36.8)
1.000–3.000 €	106 (52.7)
≥ 3.000 €	21 (10.4)
Having children	98 (48.8)
Visited a specialist for psychological problems in the past	60 (29.9)
Ever received treatment for psychological reasons	11 (5.5)
Psychotherapy in the past	45 (22.4)
Being abused during childhood	49 (24.4)
Being abused during adulthood	48 (23.9)
Being abused during a health service visit	48 (23.9)
Stressful event during last year	82 (40.8)
EPDS scale, mean (SD)	5.2 (4.2)
PSS-10, mean (SD)	14.8 (6.4)
Active positive coping, mean (SD)	26.6 (4.9)
Behavioural disengagement, mean (SD)	4.1 (1.4)
Substance abuse, mean (SD)	2.2 (0.7)
Seeking support, mean (SD)	10.9 (3.2)
Religion, mean (SD)	4.2 (1.9)
Humor, mean (SD)	4 (1.4)
Avoidance, mean (SD)	6.9 (2)
Express negative feelings, mean (SD)	7.3 (2.1)
State, mean (SD)	39.0 (10.5)
Trait, mean (SD)	41.4 (7.4)

**Table 2:** Confirmatory factor analysis indexes

	5-factor model
MLRχ2	250.13
*df*	160
MLRχ2/ *df*	1.56
RMSEA	0.05
CFI	0.92
TLI	0.91
SRMR	0.06

*Note.* RMSEA = root-mean-square error of approximation; CFI = comparative fit index (CFI); TLI = Tucker Lewis Index (TLI); SRMR = Standardized Root Mean Square Residual

**Table 3:** Descriptives of TES-A items, item-total correlations and Cronbach’s a

Factor	Item	Mean (SD)	Corrected Item-Total Correlation	Cronbach’s Alpha if Item Deleted	Cronbach’s Alpha
Anticipation of trauma	a	2.87 (0.80)	0.47	0.63	0.70
	b	1.67 (0.86)	0.50	0.62	
	c	1.63 (0.75)	0.41	0.67	
	d	2.12 (0.85)	0.53	0.59	
Intrusion	1	1.84 (0.90)	0.55	0.73	0.77
	2	1.29 (0.61)	0.38	0.78	
	3	1.81 (0.90)	0.68	0.68	
	4	1.70 (0.89)	0.64	0.70	
	5	1.36 (0.64)	0.50	0.75	
Avoidance	6	1.43 (0.77)	0.46	0.65	0.71
	7	1.20 (0.57)	0.50	0.68	
	8	1.76 (0.86)	0.46	0.65	
Resignation	9	2.57 (1.06)	0.50	0.70	0.78
	10	1.78 (1.01)	0.48	0.71	
	11	1.21 (0.50)	0.54	0.75	
	12	1.16 (0.46)	0.58	0.74	
Hyperstimulation	14	2.05 (0.95)	0.65	0.75	0.81
	15	2.00 (0.94)	0.56	0.79	
	16	2.08 (0.97)	0.68	0.73	
	17	2.15 (0.95)	0.61	0.77	

**Table 4:** Descriptive statistics for TES-A factors and their correlations between them

		Minimum	Maximum	Mean (SD)	Median (IQR)	Pearson’s correlation coefficients				
						1	2	3	4	5
1	Anticipation of trauma	4.00	15.00	8.3 (2.4)	8 (7 ─ 10)	1.00	0.62***	0.36***	0.24**	0.28***
2	Intrusion	5.00	18.00	8 (2.9)	7 (6 ─ 10)		1.00	0.49***	0.32***	0.48***
3	Avoidance	3.00	10.00	4.4 (1.6)	4 (3 ─ 5)			1.00	0.27***	0.27***
4	Resignation	4.00	14.00	6.7 (2.2)	6 (5 ─ 8)				1.00	0.48***
5	Hyper stimulation	4.00	16.00	8.3 (3)	8 (6 ─ 11)					1.00

*p < .05; **p < .01; ***p < .001

**Table 5:** Pearson’s correlation coefficients of TES-A factors with EPDS, PSS, Brief-cope and STAI scales

	Anticipation of trauma	Intrusion	Avoidance	Resignation	Hyper stimulation
EPDS scale	0.27***	0.45***	0.28***	0.52***	0.56***
PSS-10	0.25***	0.42***	0.20**	0.45***	0.51**
Active positive coping	0.11	− 0.10	− 0.01	0.01	− 0.03
Behavioural disengagement	0.11	0.19**	0.20**	0.25***	0.26***
Substance abuse	0.05	0.05	0.11	0.11	0.13
Seeking support	0.04	− 0.05	− 0.09	− 0.03	0.02
Religion	− 0.11	− 0.14	− 0.07	0.03	0.08
Humor	0.12	− 0.02	− 0.02	0.02	0.06
Avoidance	0.25***	0.25***	0.14	0.16*	0.25***
Express negative feelings	0.26***	0.19**	0.10	0.26***	0.24***
State	0.38***	0.56***	0.35***	0.48***	0.53***
Trait	0.36***	0.50***	0.25***	0.35***	0.51***

*p < .05; **p < .01; ***p < .001

## Data Availability

Upon rational demand, the corresponding author will provide the datasets used and analyzed during the current study.

## List of abbreviations

CFA: Confirmatory factor analysis
CFI: Comparative fit index
COPE: Coping Orientations to Problems Experienced
CS: Cesarean section
EPDS: Edinburgh Postnatal Depression Scale
GrTES-A: Greek version of TES-A
PSS-10: Perceived Stress Scale
PTSD: Posttraumatic Stress Disorder
RMSEA: Root mean square error of approximation
SRMR: Standardized root mean square residual
STAI: State-Trait Anxiety Inventory
TLI: Tucker Lewis index
TES: Traumatic Event Scale
χ2/df: Chi-square by degrees of freedom ratio
